# Regulation of RNA Polymerase II Transcription Initiation and Elongation by Transcription Factor TFII-I

**DOI:** 10.3389/fmolb.2021.681550

**Published:** 2021-05-13

**Authors:** Niko Linzer, Alexis Trumbull, Rukiye Nar, Matthew D. Gibbons, David T. Yu, John Strouboulis, Jörg Bungert

**Affiliations:** ^1^Department of Biochemistry and Molecular Biology, College of Medicine, UF Health Cancer Center, Genetics Institute, Powell Gene Therapy Center, University of Florida, Gainesville, FL, United States; ^2^Comprehensive Cancer Center, School of Cancer and Pharmaceutical Sciences, King’s College London, United Kingdom

**Keywords:** RNA polymerase II, transcription regulation, TFII-I, GTF2I, transcription elongation

## Abstract

Transcription by RNA polymerase II (Pol II) is regulated by different processes, including alterations in chromatin structure, interactions between distal regulatory elements and promoters, formation of transcription domains enriched for Pol II and co-regulators, and mechanisms involved in the initiation, elongation, and termination steps of transcription. Transcription factor TFII-I, originally identified as an initiator (INR)-binding protein, contains multiple protein–protein interaction domains and plays diverse roles in the regulation of transcription. Genome-wide analysis revealed that TFII-I associates with expressed as well as repressed genes. Consistently, TFII-I interacts with co-regulators that either positively or negatively regulate the transcription. Furthermore, TFII-I has been shown to regulate transcription pausing by interacting with proteins that promote or inhibit the elongation step of transcription. Changes in TFII-I expression in humans are associated with neurological and immunological diseases as well as cancer. Furthermore, TFII-I is essential for the development of mice and represents a barrier for the induction of pluripotency. Here, we review the known functions of TFII-I related to the regulation of Pol II transcription at the stages of initiation and elongation.

## Discovery of TFII-I as an Initiator-Binding Protein

The discovery of the three eukaryotic RNA polymerases and the development of powerful *in vitro* techniques for the analysis of the transcription process initiated a large body of work that led to the identification of basal promoter elements and trans-acting proteins involved in initiating the transcription of protein-coding genes by RNA polymerase II (Pol II; Roeder and Rutter, 1969; Weil et al., 1979; Roeder, 2019; Schier and Taatjes, 2020). Earlier, most of the studies were performed using viral genes containing strong promoter elements that recruit the transcription machinery with high efficiency. A critical component in the initiation step of the transcription process by Pol II is the TFIID (transcription factor II D) complex, which is composed of the TATA-binding protein (TBP) and TBP-associated factors (TAFs; Patel et al., 2020). TATA-box-containing promoters are usually found at developmentally regulated genes and characterized by the presence of a focused transcription start site (TSS), while TATA-less promoters are often found at housekeeping genes and exhibit transcription initiation over broad regions (Bhuiyan and Timmers, 2019). At TATA-box-containing promoter regions, TBP is sufficient for the reconstitution of basal transcription *in vitro*. Among the pioneering work on Pol II transcription was the discovery of the initiator element by Smale and Baltimore (1989). The initiator is a pyrimidine-rich DNA sequence that overlaps with the sequence of the TSS and was shown to be able to direct accurate transcription in the absence of a TATA box. Transcription factor TFII-I was one of the early proteins identified to interact with the initiator and to recruit transcription complexes to TATA-less promoters (Roy et al., 1993b). Subsequent studies have shown that components of the TFIID complex, including TAF1 and TAF2, interact with the initiator as well as with downstream promoter elements (DPEs), which were discovered by the Kadonaga laboratory (Burke and Kadonaga, 1996; Patel et al., 2018; Vo Ngoc et al., 2020). However, efficient transcription of TATA-less promoters cannot be reconstituted with TFIID and the other basal transcription factors alone, suggesting that additional components are essential for the initial recruitment of TFIID or stabilization of TFIID at TATA-less promoters. Furthermore, TFII-I may act to regulate the transcription of a specific set of genes *via* the initiator and/or in response to specific environmental signals *in vivo* (Roy, 2012).

## Structure and Function of TFII-I

**FIGURE 1:**
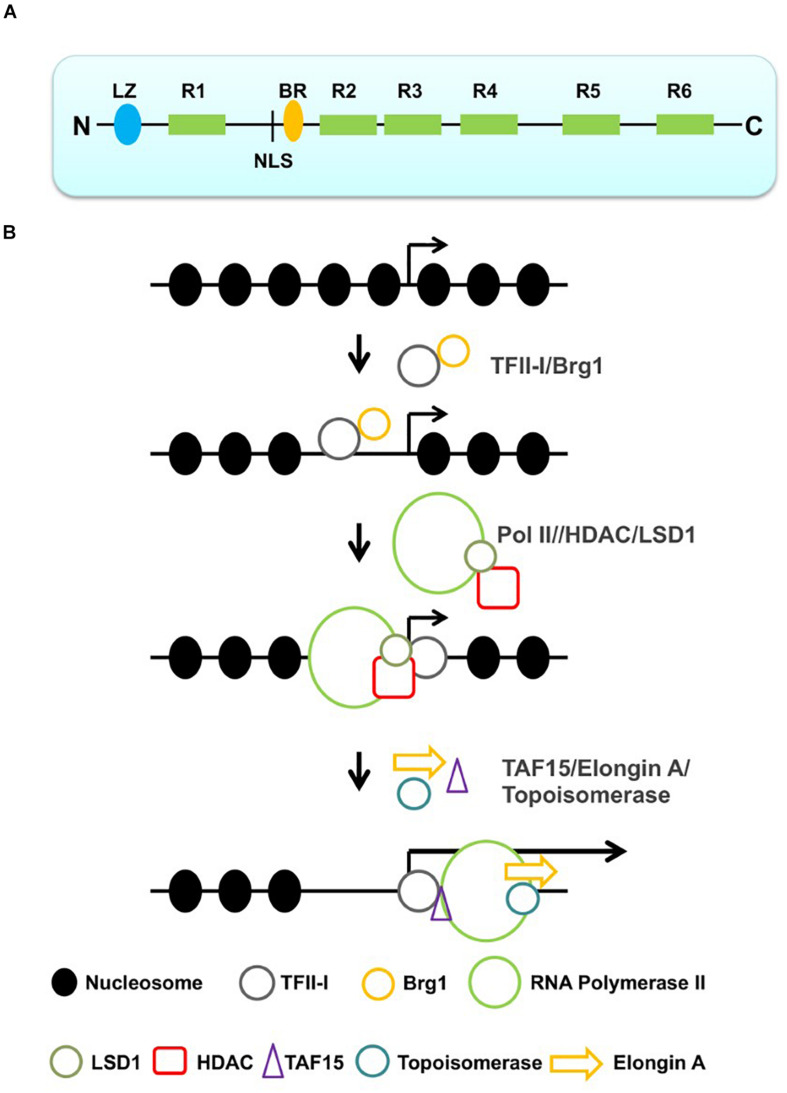
Structure of TFII-I and sequential action of TFII-I leading to the recruitment of a productive Pol II transcription complex. **(A)** Structure of TFII-I (LZ, leucine zipper; R1–R6, I-repeats; NLS, nuclear localization sequence; BR, basic region). **(B)** TFII-I interacts with the chromatin remodeler Brg1 and establishes an accessible chromatin configuration at a specific promoter. Interactions with negative co-regulators (HDAC and LSD1) keep the promoter in an accessible but inactive configuration. Dissociation of the negative co-regulators and association with positive transcription elongation factors (TAF15, topoisomerase and Elongin A) converts Pol II into a productive elongation complex.

TFII-I is an unusual transcription factor consisting of a basic region (BR) DNA-binding domain, a nuclear localization sequence (NLS), and multiple protein–protein interaction domains, including a leucine zipper (LZ) and six helix-loop-helix (HLH)-like domains, also referred to as I-repeats (R1–R6, Figure 1A; Doi-Katayama et al., 2007; Roy, 2012). TFII-I has been shown to interact not only with initiator sequences and E-boxes (HLH-binding motif, CANNTG) but also with other sequences, including the serum response element (SRE) *in vitro* (Grueneberg et al., 1997; Roy, 2012). Some of these interactions are likely mediated by other transcription factors that are associated with TFII-I. For example, TFII-I interacts with the HLH- and E-box-binding proteins USF (upstream stimulatory factor) and Myc (myelocytomatosis; Roy et al., 1991, 1993a). While cooperative interactions between TFII-I and USF activate the transcription process, the interactions between TFII-I and Myc repress transcription at the adenovirus 2 major late promoter (Ad2MLP) region. These studies on the aforementioned interactions have already provided evidence that TFII-I can function as a transcription activator or repressor depending on the interacting partner protein(s).

Since these initial studies, subsequent work has demonstrated that TFII-I is a multifunctional protein that exerts activities in both cytoplasm and nucleus (Roy, 2012). In the cytoplasm, TFII-I inhibits agonist-induced calcium entry by the transient receptor potential cation channel subfamily C member 3 (TRPC-3; Caraveo et al., 2006). In the nucleus, TFII-I functions as an activator or repressor of gene expression and also plays a role in translesion DNA repair (Roy, 2012; Fattah et al., 2014). Furthermore, TFII-I shuttles between the cytoplasm and the nucleus in response to specific signals, which is regulated by tyrosine phosphorylation, and several studies have shown that TFII-I facilitates the nuclear import of transcription factors, including members of the nuclear factor NF-κb family (Ashworth and Roy, 2007; Roy, 2012).

Alternative splicing generates four isoforms of TFII-I (α, β, Δ, and γ), which are expressed in a ubiquitous or cell type-specific manner (Roy, 2012). The Δ-isoform is ubiquitously expressed and shuttles between the cytoplasm and the nucleus. The α- and β-isoforms contain additional but exclusive short exons upstream of the NLS. The γ-isoform contains both alternative exons found in the α- and β-isoforms, and is predominantly expressed in neuronal tissues. In addition to these isoforms, there are also TFII-I-related genes expressed in humans and mice (Roy, 2012). One of these genes, *GTF2IRD1*, is located in close proximity to the *GTF2I* gene and encodes BEN (binding factor of early enhancer) (Bayarsaihan and Ruddle, 2000). Williams–Beuren syndrome (WBS) is characterized by haploinsufficiency of a relatively large genomic region on human chromosome 7 encompassing *GTF2I* and *GTF2IRD1* genes (Pober, 2010). Individuals afflicted by this large genomic deletion exhibit craniofacial, cardiovascular, and neurologic defects. Some aspects of this compound genetic disease are recapitulated in mice lacking TFII-I or BEN (Tassabehji et al., 2005; Enkhmandakh et al., 2009).

## TFII-I Function in Proliferating Cells and Cancer

TFII-I has been implicated in a variety of diseases, including neurological abnormalities. This has recently been reviewed by Roy (2017) and will not be repeated here. The role of TFII-I in the proliferation of cells and cancer will be briefly reviewed here as it relates to the multiple functions associated with this complex transcription factor. TFII-I regulates the proliferation of cells in response to serum and mitogenic signals (Roy, 2012). Phosphorylation of TFII-I by tyrosine kinases like Src (Sarcoma) and Bruton’s tyrosine kinase (BTK) leads to nuclear translocation and activation of serum response genes, including the c-fos gene (Cheriyath et al., 2002). It has also been shown that TFII-I is sequestered in the cytoplasm by the p190 Rho GTPase-activating protein through the FF domain, which is characterized by the presence of two conserved phenylalanine (FF) residues (Jiang et al., 2005). Phosphorylation of the FF domain by Src in response to serum releases TFII-I and leads to the translocation of TFII-I to the nucleus and activation of serum response genes. One of the target genes of TFII-I in the nucleus is glucose-regulated protein 78 (GRP78), a protein chaperone involved in endoplasmic reticulum (ER) stress (Hong et al., 2005). GRP78 plays an essential role in the prosurvival machinery, and high-level expression of GRP78 is associated with drug resistance, carcinogenesis, and metastasis (Ibrahim et al., 2019). TFII-I also regulates genes involved in DNA repair and is directly involved in DNA translesion repair (Roy, 2012; Fattah et al., 2014), thus leading to genome stability, which may be an important function during the proliferation of cells.

Mutations in TFII-I are associated with a number of different tumors, including T-cell lymphoma and thymus epithelial tumors (TETs; Radovich et al., 2018; Nathany et al., 2021). Point mutations in the TFII-I-coding region were found in about 6% of patients with angioimmunoblastic T-cell lymphomas (Vallois et al., 2016). A missense mutation (Leu404His) in TFII-I was found in a large number of type A and type AB thymomas (Petrini et al., 2014). This mutation was found to increase the expression of TFII-I, which may be due to the disruption of a potential destruction box (Oberndorfer and Müllauer, 2020). Previous studies have shown that TFII-I is subjected to ubiquitylation and proteasomal degradation in response to genotoxic stress (Desgranges et al., 2005). Increased expression of TFII-I in thymomas is consistent with its role in activating genes involved in proliferation (Roy, 2017). However, TFII-I was not found to be overexpressed in the most aggressive forms of thymomas (Petrini et al., 2014; Oberndorfer and Müllauer, 2020). This is interesting in light of the fact that previous studies have shown that TFII-I represents a roadblock in the generation of induced pluripotent stem cells (IPSCs; Yang et al., 2014). This suggests that although TFII-I contributes to the proliferation of cells by modulating the expression of cell cycle genes, it prevents dedifferentiation of cells. This could be due to the fact that TFII-I also regulates genes that constitute cell identity.

## TFII-I and Establishment of Chromatin Domains

Transcription factors impact gene expression in many different ways. They can bind to and act in close proximity to the genes they regulate, or they act at a distance by binding to enhancer or insulator sequences. Recent advances in our understanding of the organization of genes within the nucleus demonstrate that the genome is organized in defined topologically associating domains (TADs) that are often multiple Mb-long and are characterized by frequent chromosomal interactions within TADs and limited contacts between TADs (Sun et al., 2019). TADs are separated from each other by boundary elements that interact with CCCTC-binding protein (CTCF) and cohesin and/or condensin complexes. Within TADs, genes are organized in insulated neighborhoods in which one or several, sometimes co-regulated, genes and corresponding enhancer elements are localized. At least a subset of insulated neighborhoods is established by dimerization of CTCF proteins that interact with insulator sequences flanking the neighborhoods (Luo et al., 2020).

The genome-wide analysis of TFII-I-chromatin interactions revealed that TFII-I associates with active and with repressed genes (Makeyev et al., 2012; Fan et al., 2014). While it is evident that TFII-I regulates genes directly *via* interactions with specific DNA elements, only 8% of genomic binding sites for TFII-I in IPSCs correspond to nearby genes that change expression in response to TFII-I depletion (Makeyev et al., 2012). Furthermore, a vast majority of genes that changed expression upon TFII-I depletion did not contain binding sites for TFII-I, suggesting they are regulated indirectly. TFII-I interacts with CTCF and often associates with genomic sites occupied by CTCF (Peña-Hernández et al., 2015). Furthermore, TFII-I peaks also overlap with peaks for Rad21, a component of the cohesin complex, and pull-down experiments identified subunits of cohesin and condensin [structural maintenance of chromosomes (SMC) 2, 3, and 6] as interaction partners of TFII-I in human erythroleukemia K562 cells (Fan et al., 2014; Kim et al., 2015). These data point to the possibility that TFII-I exerts part of its function by assisting CTCF and cohesins in the establishment of TADs and/or insulated neighborhoods.

## TFII-I Represses and Activates Transcription by Pol II

Pull-down experiments identified chromatin remodeling complexes, particularly Brg1, histone deacetylases (HDACs), lysine-specific demethylase 1 (LSD1), topoisomerases, and transcription elongation factors as TFII-I-interacting proteins, suggesting diverse functions of TFII-I during the regulation of transcription (Fan et al., 2014; Adamo et al., 2015). LSD1 removes methyl groups from H3K4, and H3K4 methylation is associated with transcriptionally active or permissive chromatin (Meier and Brehm, 2014). Thus, TFII-I likely inhibits transcription through interactions with HDACs and LSD1. Genome-wide TFII-I peaks are often associated with binding sites for related (e.g., USF) or unrelated transcription factors (e.g., E2F and CTCF) (Makeyev et al., 2012; Fan et al., 2014; Peña-Hernández et al., 2015). Consistent with these findings, TFII-I interacts with E-box-binding proteins (e.g., USF and cMyc) as well as with E2F transcription factors and CTCF (Roy et al., 1991, 1993a; Fan et al., 2014; Peña-Hernández et al., 2015; Shen et al., 2018). Thus, a large fraction of TFII-I-binding events in the context of chromatin may be mediated by other DNA-binding transcription factors rather than direct interactions of TFII-I with DNA.

As mentioned before, TFII-I interacts with negative and positive co-regulators. At some gene loci, perhaps at those involved in stress response or in cell cycle control, TFII-I may play negative and positive roles at different stages of induction (Figure 1B). It is conceivable that TFII-I recruits the Brg1 chromatin remodeling complex to these genomic loci and establishes short regions of accessibility. These regions may further associate with other repressor or co-repressor proteins, e.g., repressor E2Fs, HDACs, and LSD1. Binding of these components will keep regulatory regions in an accessible but inactive configuration. Upon specific signals, e.g., growth factors, stress, or cell cycle progression, the inhibitory proteins leave the promoter from the DNA and TFII-I recruits positive factors that mediate the recruitment of Pol II or stimulate the elongation step of transcription. The poised state may also involve a paused RNA polymerase, which is outlined in the next section.

## TFII-I Regulates the Transition From Transcription Pausing to Elongation

At a subset of genes, Pol II pauses near the TSS and several activities have been identified to mediate the transition from pausing to productive elongation (Gonzales et al., 2021). At mRNA genes, Pol II pauses to allow capping of the 5′end of the RNA. This pausing is mediated by negative elongation factor (NELF) and 5,6-dichloro-1-β-d-ribofuranosylbenzimidazole (DRB) sensitivity-inducing factor (DSIF; Yamaguchi et al., 2013; Schier and Taatjes, 2020). DRB is a nucleoside homolog that inhibits the elongation step of transcription. DSIF interacts with the initially transcribed RNA and recruits NELF. Pol II consists of a relatively unstructured C-terminal domain (CTD) that contains a repeated heptapeptide sequence (Harlen and Churchman, 2017). Within the repeated heptapeptide sequence, there are several serine (S) residues that are subject to phosphorylation during transcription (Harlen and Churchman, 2017; Schier and Taatjes, 2020). Upon the initiation of transcription, the basal transcription factor TFII-H phosphorylates S5 (Schier and Taatjes, 2020). This phosphorylation event disrupts interactions with basal transcription factors and the mediator–co-activator complex and promotes interactions with DSIF, NELF, and the capping complex. Interactions of NELF with Pol II prevent the association with positive elongation factor TFIIS and with the RNA polymerase-associated factor (PAF) complex (Vos et al., 2018). The binding of NELF also leads to an inactive conformation of Pol II that prevents translocations and base pairing of nucleotides in the active site. After capping, positive transcription elongation factor b (pTEFb) phosphorylates DSIF, NELF, and the CTD residue S2 (Yamaguchi et al., 2013; Schier and Taatjes, 2020). S2P assists in recruiting the PAF complex as well as RNA-processing factors. Phosphorylation of DSIF converts it from a negative to a positive elongation factor. Phosphorylation of NELF causes its dissociation from the transcription complex allowing interactions of Pol II with PAF and TFIIS, and transitioning from the paused to the elongation-competent form (Yamaguchi et al., 2013; Vos et al., 2018; Schier and Taatjes, 2020).

The Elongin complex has been shown to regulate Pol II transcription elongation activity (Conaway and Conaway, 1999). Studies by the Conaway laboratory demonstrated that Elongin A associates with genes at regions occupied by S5P-modified Pol II (Kawauchi et al., 2013). Furthermore, *in vitro* studies demonstrated that Elongin A stimulates the elongation step of transcription (Conaway and Conaway, 1999). Recent genome-wide analysis of Elongin A-deficient cells did not reveal strong defects in overall Pol II transcription elongation rates but showed increased accumulation of Pol II at TSSs, suggesting that Elongin A regulates the transition from pause to transcription elongation (Ardehali et al., 2020; Wang et al., 2020). Interestingly, the genome-wide occupancy data suggest that Elongin A is preferentially recruited to sites upstream of the TSS and to enhancer elements. This could indicate that Elongin A is recruited by sequence-specific transcription activators that bind promoters and/or enhancers. Furthermore, RNA-seq data show that Elongin A deficiency only affects the expression of a small set of genes (Ardehali et al., 2020; Wang et al., 2020).

TFII-I has been shown to interact with NELF as well as Elongin A (Fan et al., 2014; McCleary-Wheeler et al., 2020). It appears that these interactions play a role in the inducible expression of specific genes. For example, both Elongin A and TFII-I are important for maximal stress-dependent induction of the activating transcription factor 3 (ATF3) gene (Fan et al., 2014). ATF3 is induced in response to a variety of cellular stress signals, including ER stress (Ku and Cheng, 2020). TFII-I has previously been implicated in gene regulation following ER stress (Parker et al., 2001). Elongin A was shown to interact with the transcribed region of ATF3, and this interaction is increased upon the induction of ER stress (Fan et al., 2014). Pol II peaks are associated with a putative enhancer element located far upstream of the gene and with the promoter. TFII-I interacts immediately downstream of the Pol II peak at the enhancer element. Upon stress, increased transcription is not only observed at the promoter but also downstream of the enhancer (Fan et al., 2014). Importantly, increased transcription is associated with enhanced recruitment of Elongin A to the ATF3 promoter region. TFII-I also interacts with topoisomerases that remove torsional stress of the DNA during the elongation step of transcription. These data suggest that TFII-plays a positive role in recruiting and/or modulating the activity of positive transcription elongation factors.

As mentioned before, TFII-I was shown to interact with the insulator protein CTCF and to regulate genes in response to metabolic stress (Peña-Hernández et al., 2015). This is interesting in light of previous studies showing that CTCF, in addition to serving as an insulator-binding protein (IBP), regulates transcriptional pausing (Shukla et al., 2011; Herrera Paredes et al., 2013). Binding of CTCF to the proximal promoter has been shown to increase the pausing index. However, CTCF has also been implicated in the positive regulation of the elongation step of transcription by mediating the recruitment of pTEFb (Laitem et al., 2015). Ablating TFII-I expression led to a reduction of CTCF binding at specific promoters concomitant with the reduced expression of these genes (Marques et al., 2014; Peña-Hernández et al., 2015). Moreover, TFII-I deficiency was not only associated with reduced CTCF binding but also with an impaired recruitment of CDK8 and a reduction of Pol II S5P at CTCF target genes (Marques et al., 2014; Peña-Hernández et al., 2015). Thus, at certain genes, TFII-I cooperates with CTCF in mediating transcription likely by modulating early Pol II transcription initiation events. In *Drosophila*, other IBPs have also been shown to modulate Pol II pausing at distantly located genes. This is mediated by their common cofactor CP190 (Liang et al., 2014).

**FIGURE 2:**
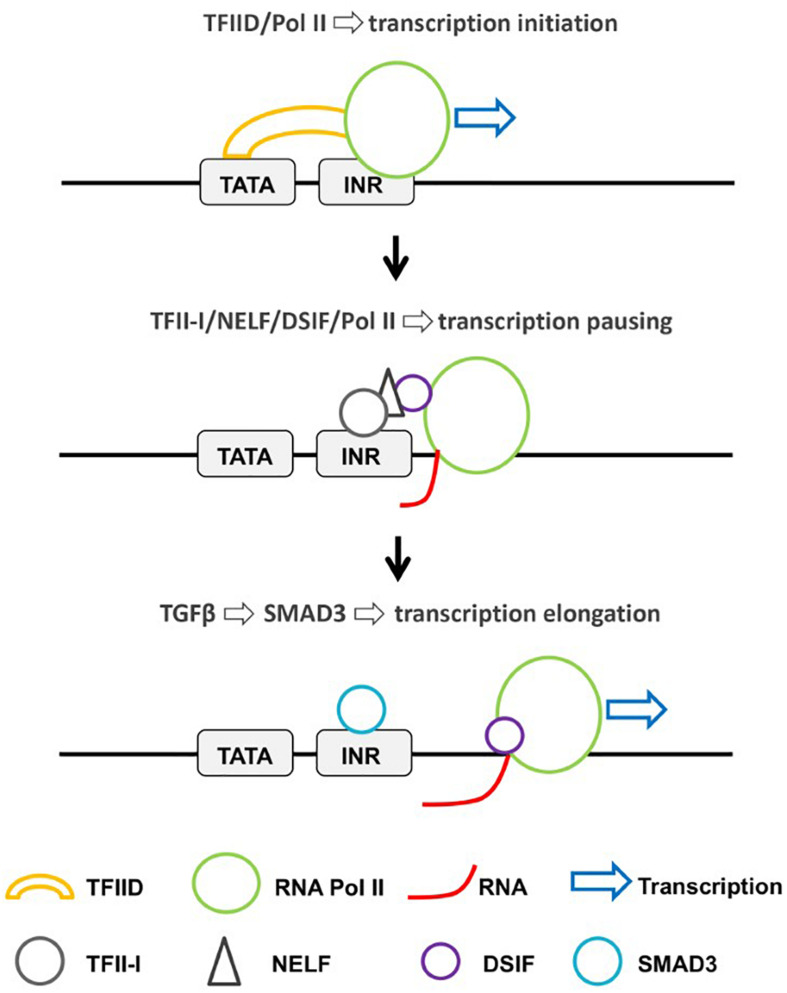
Regulation of Pol II recruitment and transcriptional pausing by TFFI-I. At a subset of TGF-β-inducible genes, TFII-I interacts with NELF and DSIF at a post-initiation step and prevents the release of Pol II from the paused state (McCleary-Wheeler et al., 2020). TGF-β signaling increases the nuclear localization of SMAD3, which displaces TFII-I and converts Pol II into a productive elongation complex.

The above-discussed studies implicate TFII-I in the positive regulation of the elongation step of transcription. A recent study implicates TFII-I in the negative regulation of genes induced by TGF-β (McCleary-Wheeler et al., 2020). At a subset of TGF-β-induced genes, Pol II is paused downstream of the TSS (Figure 2). The paused Pol II is associated with NELF and DSIF. TFII-I was shown to bind at the TSS of these genes and to interact with NELF and DSIF. The authors propose that small mothers against decapentaplegic 3 (SMAD3), induced by transforming growth factor β (TGF-β), displaces TFII-I from the TSS, thus dissociating NELF and converting Pol II into an elongation-competent form. It is an intriguing idea that TFII-I may interact with TSSs after Pol II initiates transcription to regulate the pausing step. This is consistent with the fact that TFIID is the major protein complex recruiting Pol II to basal promoter elements, and reinforces the idea that TFII-I may regulate transcription at a step post Pol II recruitment.

Recently, it was shown that NELF forms nuclear condensates in response to stress (Rawat et al., 2021). Formation of nuclear condensates is dependent on the presence of an intrinsically disordered region (IDR) and is driven by dephosphorylation and sumoylation of NELF. This process increases the recruitment of NELF to promoters and causes transcription repression. TFII-I has previously been shown to interact with TAF15, one of the FET (Fus/EWS/TAF15) proteins (Fan et al., 2014). FET proteins contain IDRs that drive phase separation (Wang et al., 2018). Loci-specific phase separation mediated by Fet proteins recruits RNA Pol II to promoters and activates transcription (Wei et al., 2020; Zuo et al., 2021). Thus, TFII-I may regulate transcription negatively through NELF-driven phase separation, or positively through TAF15-driven phase separation.

## Conclusion and Outlook

Because of its unique structure, its multiple functions in thenucleus and cytoplasm, and its implication in cancer as well as neurological and immunological disorders, there is interest and a significant requirement to elucidate the mechanisms by which TFII-I affects gene expression patterns and other cellular functions during development and differentiation. It is clear that TFII-I is a DNA-binding protein that interacts with co-regulators to positively or negatively affect the transcription of specific target genes, but many aspects of TFII-I function remain enigmatic. It seems that a fraction of TFII-I chromatin associations is mediated by interactions with other DNA-binding proteins, including associations with E-box sequences together with HLH proteins, associations with E2F sites together with E2F transcription factors, and associations with insulator sequences together with CTCF and perhaps components of cohesin. In addition, TFII-I interacts with a variety of proteins that regulate different steps in the process of transcription, including histone-modifying enzymes, topoisomerases, and transcription elongation factors. Due to its relatively large size and the presence of multiple protein–protein interaction domains, it is possible that TFII-I functions as a hub to regulate the coordinated recruitment of activities involved in gene regulation. Its putative involvement in the creation of chromatin domains and insulated chromatin neighborhoods will be an exciting focus of future research. Furthermore, the recent findings implicating TFII-I in regulating the elongation step of transcription suggest that it will be important to determine its function in response to stress or other signals. As mentioned before, TFII-I has been shown to interact with TAF15, which is capable of forming phase-separated domains (Fan et al., 2014; Wang et al., 2018). Recent evidence suggests that the transcription of highly expressed genes is regulated by phase-separated super-enhancers (Ishov et al., 2020). The elongation step of transcription occurs away from these domains and toward RNA processing domains. It will be interesting to investigate if TFII-I is involved in the formation of phase-separated transcription initiation domains and/or in associations of specific genes with RNA processing compartments.

## Author Contributions

JB, JS, RN, NL, and AT discussed the contents and outline of the review. All authors contributed to the writing and generation of figures.

## Conflict of Interest

The authors declare that the research was conducted in the absence of any commercial or financial relationships that could be construed as a potential conflict of interest.
